# Supplementary material for: Multi-Algorithm Analysis Reveals Pyroptosis-Linked Genes as Pancreatic Cancer Biomarkers
Source: Cancers (Basel). 2024 Jan 15;16(2):372. doi: 10.3390/cancers16020372 (PMC10814254; doi:10.3390/cancers16020372)
Supplement: Supplementary file 1 [file cancers-16-00372-s001.zip › cancers-2723643-supplementary.pdf]

Multi-Algorithm Analysis Reveals Pyroptosis-Linked Genes as Pancreatic Cancer Biomarkers

Kangtao Wang, Shanshan Han, Li Liu, Lian Zhao, Ingrid Herr

Table of Content

Supplemental information S1. LDA Core Code

Supplemental information S2. Example of Nomogram

Figure S1. Survival Prediction Results for Two Sample Patients in the Nomogram.

Table S1. Gene Expression, Risk Score, and Risk Group for Two Patients.

Table S2. Links to Clinicopathological Data and Transcriptome Data Acquisition

Table S3. RT-qPCR Primer Sequences for RT-qPCR

Table S4. List of Pyroptosis-Related Genes

Supplemental information S1. LDA core code

```
# improved version from our previous work, https://github.com/yanwen0614/Medicine-Bibliometric-Analysis
import logging
import os
import pickle
import pandas as pd
from nltk.tokenize import RegexpTokenizer
from nltk.stem.porter import PorterStemmer
from gensim import corpora, models
from gensim.models.ldamulticore import LdaMulticore

logging.basicConfig(level=logging.INFO, format='%(asctime)s - %(name)s - %(levelname)s - %(message)s')
logger = logging.getLogger(__name__)


def dump_pickle(filepath, year):
    corpus = []
    tokens = []

    df = pd.read_csv(filepath, sep='\t', encoding="utf-8-sig", error_bad_lines=False)
    df = df.dropna(subset=["Abstract"])
    df["year"] = pd.to_numeric(df["year"], errors="coerce")
    df = df[df["year"] < year]

    tokenizer = RegexpTokenizer(r'[A-Za-z]+')
    en_stop = [str(i).strip() for i in open("stopwords.txt", encoding="utf-8-sig")]

    p_stemmer = PorterStemmer()

    logging.info("Processing documents...")
    for i, line in enumerate(df["Abstract"]):
        if i % 1000 == 0:
            logging.info(f"{i} documents processed")
        raw = line.lower()
        tokens.append([p_stemmer.stem(word) for word in tokenizer.tokenize(raw) if word not in en_stop])

    dictionary = corpora.Dictionary(tokens)
    texts = [dictionary.doc2bow(text) for text in tokens]

    logging.info("Saving data to pickle files...")
```

```
    pickle.dump(texts, open("text_dtm.pickle", "wb"))
    pickle.dump(dictionary, open("dictionary.pickle", "wb"))


def load_pickle():
    texts = pickle.load(open("text_dtm.pickle", "rb"))
    dictionary = pickle.load(open("dictionary.pickle", "rb"))
    return texts, dictionary


def create_lda(num_topics, filename, year):
    dump_pickle(filename, year)
    texts, dictionary = load_pickle()

    lda = LdaMulticore(corpus=texts, iterations=1000, id2word=dictionary,
num_topics=num_topics,
                       passes=300, per_word_topics=True)

    lda.save(f"./ldamd/{num_topics}tpc-{filename[9:18]}")
    return lda


def save_topic_words(lda):
    num_topics = lda.num_topics
    topic_words = pd.DataFrame(columns=[i for i in range(1, 11)])

    for i in range(num_topics):
        topic_words.loc[i] = [word for word, _ in lda.show_topic(i)]

    topic_words.to_csv("./newdata/topic_words.csv", index=False)


def get_document_topics(lda, texts):
    tpc1 = []
    tpc2 = []

    for text in texts:
        tpc = lda.get_document_topics(text)
        tpc = sorted(tpc, key=lambda x: -x[1])
        tpc1.append(tpc[0][0])
        tpc2.append(tpc[1][0] if len(tpc) > 1 else lda.num_topics)

    return tpc1, tpc2
```

```
def generate_graph(tpc1, tpc2, num_topics, filename):
    from collections import Counter
    import networkx as nx

    CC = Counter(tpc1)
    G = nx.Graph()

    for i in range(num_topics):
        G.add_node(i, num=CC[i])

    edges_list = [(i, j) for i, j in zip(tpc1, tpc2) if j < num_topics]
    G.add_edges_from(edges_list, weight=0)

    for i, j in zip(tpc1, tpc2):
        if j >= num_topics:
            continue
        G.edges[i, j]["weight"] += 1

    nx.write_graphml(G, filename.replace(".csv", ".graphml"), encoding="utf8")
    return G
    def main(year):
    filename = "total.csv"

    if not os.path.exists("./newdata"):
        os.makedirs("./newdata")
    if not os.path.exists("./ldamd"):
        os.makedirs("./ldamd")

    lda = create_lda(50, filename, year)

    save_topic_words(lda)
    logging.info("LDA Finished")
    logging.info("Calculating topics for each document")

    texts, _ = load_pickle()
    tpc1, tpc2 = get_document_topics(lda, texts)

    logging.info("Generating graph")
    generate_graph(tpc1, tpc2, 50, filename)
    if name == "main":
    year = 2023
    main(year)
```

Supplemental information S2. Example of Nomogram

1. Please visit the website: nomogram-uniheidelberg.shinyapps.io/DynNomapp/.
2. We have chosen two patients as examples, and the gene expressions for these two patients are shown in Table S1.

Table S1. Gene expression, risk score, and risk group for two patients.

	BHLHE40	APOL1	IL18	BIRC3	Risk Score	Risk group
**Patient 1**	7.589151	6.769928	5.281003	4.733575	4.12733661	High risk
**Patient 2**	5.306221	4.669242	2.259798	1.459015	2.14861809	Low risk

3. Subsequently, inputting the clinical information of the patients, assuming both are 53 years old, female, with tumor staging at stage 1, and Grade 1, into our nomogram, we can obtain predictions for the patients' survival time and other relevant information, Figure S2.. In the future, we aspire to incorporate additional features, such as drug sensitivity for different patient groups, However, it's worth noting that this aspect is still under research and will be refined in the future.

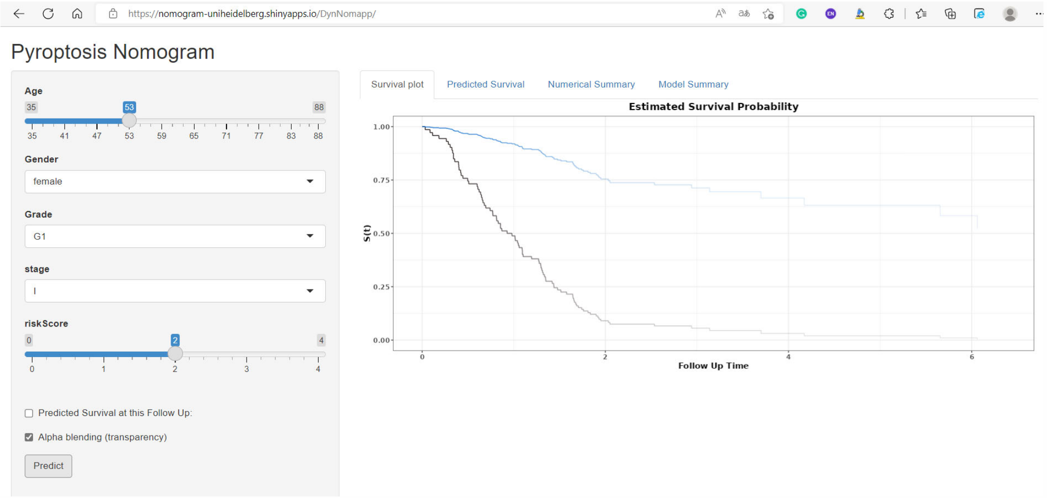

Figure S1. Survival prediction results for two sample patients in the nomogram.

Table S2. Links to Clinicopathological Data and Transcriptome Data Acquisition

Name	Sample sizes	Web sites
**TCGA-PAAD**	177	https://portal.gdc.cancer.gov/
**ICGC-PACA-AU**	91	https://dcc.icgc.org/projects/PACA-AU
**ICGC-PACA-CA**	213	https://dcc.icgc.org/projects/PACA-CA
**GSE71729**	105	https://www.ncbi.nlm.nih.gov/geo/query/acc.cgi?acc=GSE71729
**GSE62452**	66	https://www.ncbi.nlm.nih.gov/geo/query/acc.cgi?acc=GSE62452
**GSE57495**	63	https://www.ncbi.nlm.nih.gov/geo/query/acc.cgi?acc=GSE57495
**GSE21501**	192	https://www.ncbi.nlm.nih.gov/geo/query/acc.cgi?acc=GSE21501
**GSE102238**	50	https://www.ncbi.nlm.nih.gov/geo/query/acc.cgi?acc=GSE102238
**E-MTAB-6134**	287	https://www.ebi.ac.uk/arrayexpress/experiments/E-MTAB-6134/

Table S3. Primer Sequences for RT-qPCR

Gene	Sequence	TM	Position
**GAPDH forward**	CATGGGTGTGAACCATGAGAA	62	NM_001256799.3 : 573-593
**GAPDH reversed**	GGTCATGAGTCCTTCCACGAT	64	NM_001256799.3 : 685-705
**BHLHE40 forward**	CAGCCGTGCTTCAAAAGTGACC	68	NM_003670.3 : 1054-1075
**BHLHE40 reversed**	AAGCTGCATCCGGTTCTTTTTT	62	NM_003670.3 : 1143-1164
**GBP1 forward**	AGAATGAGAATGAGGTTGAGG	60	NM_002053.3 : 547-567
**GBP1 reversed**	GTCCATCTGCTTCCAAGTC	58	NM_002053.3 : 630-648
**IL18 forward**	CTGACTGTAGAGATAATGCACCCC	72	NM_001243211.2 : 400-423
**IL18 reversed**	TGATGTTATCAGGAGGATTCATTTC	68	NM_001243211.2 : 546-570
**ANXA2 forward**	ACTGTTCACGAAATCCTGTG	58	NM_001002857.2 : 143-162
**ANXA2 reversed**	CCCGCTCAGCATCAAAGTTA	60	NM_001002857.2 : 229-248
**ANXA1 forward**	AGAGGAACTGAAGAGAGATCTG	64	NM_000700.3 : 545-566
**ANXA1 reversed**	TTCAACTCCAGGTCCAGAAC	60	NM_000700.3 : 825-844
**TRIM31 forward**	AAGGAAGAACGCAATCAG	52	NM_007028.5 : 290-307
**TRIM31 reversed**	TCGCAGAAATAGTGGAAC	52	NM_007028.5 : 413-430
**BIRC3 forward**	CTTTGCCTGTGGTGGAAAAT	58	NM_001165.5 : 3376-3395
**BIRC3 reversed**	ACTTGCAAGCTGCTCAGGAT	60	NM_001165.5 : 3576-3595
**APOL1 forward**	TGGACTACGGAAAGAAGTGGT	62	NM_001136540.2 : 828-848
**APOL1 reversed**	CCTCCTTCAATTTGTCAAGGCTT	66	NM_001136540.2 : 883-905
**EPHA2 forward**	GTATGGCAAAGGGTGGGACC	64	NM_004431.5 : 278-297
**EPHA2 reversed**	ACGTTGCACACGGAGTACAT	60	NM_004431.5 : 333-352
**TCEA3 forward**	AAGAGCACGGACATGAAGTACC	66	NM_003196.3 : 797-818
**TCEA3 reversed**	CTCTGCCGTCATCTTGGCTA	62	NM_003196.3 : 915-934 bp:119
**BNIP3 forward**	TCCAGCCTCGGTTTCTATTT	58	NM_004052.4 : 213-232
**BNIP3 reversed**	AGCTCTTGGAGCTACTCCGT	62	NM_004052.4 : 282-301

Table S4. List of Pyroptosis-Related Genes

Gene name or symbol	Full name or description	Gene type
**ABL1**	ABL Proto-Oncogene 1, Non-Receptor Tyrosine Kinase	Protein Coding
**ACE2**	Angiotensin Converting Enzyme 2	Protein Coding
**ADAMTS9-AS2**	ADAMTS9 Antisense RNA 2	RNA Gene
**ADORA1**	Adenosine A1 Receptor	Protein Coding
**ADORA2A**	Adenosine A2a Receptor	Protein Coding
**ADORA2B**	Adenosine A2b Receptor	Protein Coding
**ADORA3**	Adenosine A3 Receptor	Protein Coding
**AGER**	Advanced Glycosylation End-Product Specific Receptor	Protein Coding
**AIM2**	Absent In Melanoma 2	Protein Coding
**AKT1**	AKT Serine/Threonine Kinase 1	Protein Coding
**ALK**	ALK Receptor Tyrosine Kinase	Protein Coding
**ANO6**	Anoctamin 6	Protein Coding
**ANXA1**	Annexin A1	Protein Coding
**ANXA2**	Annexin A2	Protein Coding
**APAF1**	Apoptotic Peptidase Activating Factor 1	Protein Coding
**APIP**	APAF1 Interacting Protein	Protein Coding
**APOE**	Apolipoprotein E	Protein Coding
**APOL1**	Apolipoprotein L1	Protein Coding
**ASIC1**	Acid Sensing Ion Channel Subunit 1	Protein Coding
**ATF6**	Activating Transcription Factor 6	Protein Coding
**ATG3**	Autophagy Related 3	Protein Coding
**ATG7**	Autophagy Related 7	Protein Coding
**BAK1**	BCL2 Antagonist/Killer 1	Protein Coding
**BAX**	BCL2 Associated X, Apoptosis Regulator	Protein Coding
**BCL2**	BCL2 Apoptosis Regulator	Protein Coding
**BECN1**	Beclin 1	Protein Coding
**BHLHE40**	Basic Helix-Loop-Helix Family Member E40	Protein Coding
**BHLHE41**	Basic Helix-Loop-Helix Family Member E41	Protein Coding
**BIRC2**	Baculoviral IAP Repeat Containing 2	Protein Coding
**BIRC3**	Baculoviral IAP Repeat Containing 3	Protein Coding
**BNIP3**	BCL2 Interacting Protein 3	Protein Coding
**BRCC3**	BRCA1/BRCA2-Containing Complex Subunit 3	Protein Coding
**BRD4**	Bromodomain Containing 4	Protein Coding
**BSG**	Basigin (Ok Blood Group)	Protein Coding
**BST2**	Bone Marrow Stromal Cell Antigen 2	Protein Coding
**BTK**	Bruton Tyrosine Kinase	Protein Coding
**CAMP**	Cathelicidin Antimicrobial Peptide	Protein Coding
**CAPN1**	Calpain 1	Protein Coding
**CARD8**	Caspase Recruitment Domain Family Member 8	Protein Coding
**CASP1**	Caspase 1	Protein Coding
**CASP3**	Caspase 3	Protein Coding
**CASP4**	Caspase 4	Protein Coding

**CASP5**	Caspase 5	Protein Coding
**CASP6**	Caspase 6	Protein Coding
**CASP8**	Caspase 8	Protein Coding
**CASP9**	Caspase 9	Protein Coding
**CD14**	CD14 Molecule	Protein Coding
**CD274**	CD274 Molecule	Protein Coding
**CDC37**	Cell Division Cycle 37, HSP90 Cochaperone	Protein Coding
**CDK9**	Cyclin Dependent Kinase 9	Protein Coding
**CDKN2B-AS1**	CDKN2B Antisense RNA 1	RNA Gene
**CEBPB**	CCAAT Enhancer Binding Protein Beta	Protein Coding
**CGAS**	Cyclic GMP-AMP Synthase	Protein Coding
**CHI3L1**	Chitinase 3 Like 1	Protein Coding
**CHMP1A**	Charged Multivesicular Body Protein 1A	Protein Coding
**CHMP2A**	Charged Multivesicular Body Protein 2A	Protein Coding
**CHMP2B**	Charged Multivesicular Body Protein 2B	Protein Coding
**CHMP3**	Charged Multivesicular Body Protein 3	Protein Coding
**CHMP4A**	Charged Multivesicular Body Protein 4A	Protein Coding
**CHMP4B**	Charged Multivesicular Body Protein 4B	Protein Coding
**CHMP4C**	Charged Multivesicular Body Protein 4C	Protein Coding
**CHMP6**	Charged Multivesicular Body Protein 6	Protein Coding
**CHMP7**	Charged Multivesicular Body Protein 7	Protein Coding
**CHRFAM7A**	CHRNA7 (Exons 5-10) And FAM7A (Exons A-E) Fusion	Protein Coding
**CLEC5A**	C-Type Lectin Domain Containing 5A	Protein Coding
**CPTP**	Ceramide-1-Phosphate Transfer Protein	Protein Coding
**CRTAC1**	Cartilage Acidic Protein 1	Protein Coding
**CTSG**	Cathepsin G	Protein Coding
**CTSV**	Cathepsin V	Protein Coding
**CXCL8**	C-X-C Motif Chemokine Ligand 8	Protein Coding
**CYCS**	Cytochrome C, Somatic	Protein Coding
**DDX3X**	DEAD-Box Helicase 3 X-Linked	Protein Coding
**DHX9**	DExH-Box Helicase 9	Protein Coding
**DLX6-AS1**	DLX6 Antisense RNA 1	RNA Gene
**DNMT1**	DNA Methyltransferase 1	Protein Coding
**DNMT3A**	DNA Methyltransferase 3 Alpha	Protein Coding
**DNMT3B**	DNA Methyltransferase 3 Beta	Protein Coding
**DPEP1**	Dipeptidase 1	Protein Coding
**DPP8**	Dipeptidyl Peptidase 8	Protein Coding
**DPP9**	Dipeptidyl Peptidase 9	Protein Coding
**DRD2**	Dopamine Receptor D2	Protein Coding
**DUOX1**	Dual Oxidase 1	Protein Coding
**E2F4**	E2F Transcription Factor 4	Protein Coding
**EED**	Embryonic Ectoderm Development	Protein Coding
**EEF2K**	Eukaryotic Elongation Factor 2 Kinase	Protein Coding
**EGFR**	Epidermal Growth Factor Receptor	Protein Coding
**ELANE**	Elastase, Neutrophil Expressed	Protein Coding
**ELAVL1**	ELAV Like RNA Binding Protein 1	Protein Coding
**EPHA2**	EPH Receptor A2	Protein Coding

**ERP44**	Endoplasmic Reticulum Protein 44	Protein Coding
**EZH2**	Enhancer Of Zeste 2 Polycomb Repressive Complex 2 Subunit	Protein Coding
**FADD**	Fas Associated Via Death Domain	Protein Coding
**FGF21**	Fibroblast Growth Factor 21	Protein Coding
**FNDC4**	Fibronectin Type III Domain Containing 4	Protein Coding
**FNDC5**	Fibronectin Type III Domain Containing 5	Protein Coding
**FOXO3**	Forkhead Box O3	Protein Coding
**FOXP3**	Forkhead Box P3	Protein Coding
**FPR2**	Formyl Peptide Receptor 2	Protein Coding
**GAS5**	Growth Arrest Specific 5	RNA Gene
**GBP1**	Guanylate Binding Protein 1	Protein Coding
**GBP5**	Guanylate Binding Protein 5	Protein Coding
**GJA1**	Gap Junction Protein Alpha 1	Protein Coding
**GLMN**	Glomulin, FKBP Associated Protein	Protein Coding
**GPER1**	G Protein-Coupled Estrogen Receptor 1	Protein Coding
**GSDMA**	Gasdermin A	Protein Coding
**GSDMB**	Gasdermin B	Protein Coding
**GSDMC**	Gasdermin C	Protein Coding
**GSDMD**	Gasdermin D	Protein Coding
**GSDME**	Gasdermin E	Protein Coding
**GSK3B**	Glycogen Synthase Kinase 3 Beta	Protein Coding
**GSTO1**	Glutathione S-Transferase Omega 1	Protein Coding
**GZMA**	Granzyme A	Protein Coding
**GZMB**	Granzyme B	Protein Coding
**H2AB1**	H2A.B Variant Histone 1	Protein Coding
**H2AC14**	H2A Clustered Histone 14	Protein Coding
**H2AC18**	H2A Clustered Histone 18	Protein Coding
**H2AC19**	H2A Clustered Histone 19	Protein Coding
**H2AC20**	H2A Clustered Histone 20	Protein Coding
**H2AC4**	H2A Clustered Histone 4	Protein Coding
**H2AC6**	H2A Clustered Histone 6	Protein Coding
**H2AC7**	H2A Clustered Histone 7	Protein Coding
**H2AC8**	H2A Clustered Histone 8	Protein Coding
**H2AJ**	H2A.J Histone	Protein Coding
**H2AX**	H2A.X Variant Histone	Protein Coding
**H2AZ1**	H2A.Z Variant Histone 1	Protein Coding
**H2AZ2**	H2A.Z Variant Histone 2	Protein Coding
**H2BC1**	H2B Clustered Histone 1	Protein Coding
**H2BC10**	H2B Clustered Histone 10	Protein Coding
**H2BC11**	H2B Clustered Histone 11	Protein Coding
**H2BC12**	H2B Clustered Histone 12	Protein Coding
**H2BC13**	H2B Clustered Histone 13	Protein Coding
**H2BC14**	H2B Clustered Histone 14	Protein Coding
**H2BC15**	H2B Clustered Histone 15	Protein Coding
**H2BC17**	H2B Clustered Histone 17	Protein Coding
**H2BC21**	H2B Clustered Histone 21	Protein Coding
**H2BC3**	H2B Clustered Histone 3	Protein Coding
**H2BC4**	H2B Clustered Histone 4	Protein Coding

**H2BC5**	H2B Clustered Histone 5	Protein Coding
**H2BC6**	H2B Clustered Histone 6	Protein Coding
**H2BC7**	H2B Clustered Histone 7	Protein Coding
**H2BC8**	H2B Clustered Histone 8	Protein Coding
**H2BC9**	H2B Clustered Histone 9	Protein Coding
**H2BU1**	H2B.U Histone 1	Protein Coding
**H3-3A**	H3.3 Histone A	Protein Coding
**H3-3B**	H3.3 Histone B	Protein Coding
**H3C1**	H3 Clustered Histone 1	Protein Coding
**H3C10**	H3 Clustered Histone 10	Protein Coding
**H3C11**	H3 Clustered Histone 11	Protein Coding
**H3C12**	H3 Clustered Histone 12	Protein Coding
**H3C13**	H3 Clustered Histone 13	Protein Coding
**H3C14**	H3 Clustered Histone 14	Protein Coding
**H3C15**	H3 Clustered Histone 15	Protein Coding
**H3C2**	H3 Clustered Histone 2	Protein Coding
**H3C3**	H3 Clustered Histone 3	Protein Coding
**H3C4**	H3 Clustered Histone 4	Protein Coding
**H3C6**	H3 Clustered Histone 6	Protein Coding
**H3C7**	H3 Clustered Histone 7	Protein Coding
**H3C8**	H3 Clustered Histone 8	Protein Coding
**H4-16**	H4 Histone 16	Protein Coding
**H4C1**	H4 Clustered Histone 1	Protein Coding
**H4C11**	H4 Clustered Histone 11	Protein Coding
**H4C12**	H4 Clustered Histone 12	Protein Coding
**H4C13**	H4 Clustered Histone 13	Protein Coding
**H4C14**	H4 Clustered Histone 14	Protein Coding
**H4C15**	H4 Clustered Histone 15	Protein Coding
**H4C2**	H4 Clustered Histone 2	Protein Coding
**H4C3**	H4 Clustered Histone 3	Protein Coding
**H4C4**	H4 Clustered Histone 4	Protein Coding
**H4C5**	H4 Clustered Histone 5	Protein Coding
**H4C6**	H4 Clustered Histone 6	Protein Coding
**H4C8**	H4 Clustered Histone 8	Protein Coding
**H4C9**	H4 Clustered Histone 9	Protein Coding
**HDAC6**	Histone Deacetylase 6	Protein Coding
**HMGB1**	High Mobility Group Box 1	Protein Coding
**HNP1**	Hypertensive Nephropathy	Genetic Locus
**HOTTIP**	HOXA Distal Transcript Antisense RNA	RNA Gene
**HSP90AA1**	Heat Shock Protein 90 Alpha Family Class A Member 1	Protein Coding
**HSP90AB1**	Heat Shock Protein 90 Alpha Family Class B Member 1	Protein Coding
**HUWE1**	HECT, UBA And WWE Domain Containing E3 Ubiquitin Protein Ligase 1	Protein Coding
**IFI16**	Interferon Gamma Inducible Protein 16	Protein Coding
**IFIH1**	Interferon Induced With Helicase C Domain 1	Protein Coding
**IKBKE**	Inhibitor Of Nuclear Factor Kappa B Kinase Subunit Epsilon	Protein Coding
**IL13**	Interleukin 13	Protein Coding

**IL13RA2**	Interleukin 13 Receptor Subunit Alpha 2	Protein Coding
**IL18**	Interleukin 18	Protein Coding
**IL1A**	Interleukin 1 Alpha	Protein Coding
**IL1B**	Interleukin 1 Beta	Protein Coding
**IL1RN**	Interleukin 1 Receptor Antagonist	Protein Coding
**IL27**	Interleukin 27	Protein Coding
**IL32**	Interleukin 32	Protein Coding
**IL36B**	Interleukin 36 Beta	Protein Coding
**IL36G**	Interleukin 36 Gamma	Protein Coding
**IRAK3**	Interleukin 1 Receptor Associated Kinase 3	Protein Coding
**IRF1**	Interferon Regulatory Factor 1	Protein Coding
**IRF2**	Interferon Regulatory Factor 2	Protein Coding
**IRF3**	Interferon Regulatory Factor 3	Protein Coding
**IRGM**	Immunity Related GTPase M	Protein Coding
**JUN**	Jun Proto-Oncogene, AP-1 Transcription Factor Subunit	Protein Coding
**KCNQ1OT1**	KCNQ1 Opposite Strand/Antisense Transcript 1	RNA Gene
**KLF3-AS1**	KLF3 Antisense RNA 1	RNA Gene
**LINC00958**	Long Intergenic Non-Protein Coding RNA 958	RNA Gene
**LOC102724334**	Histone H2B Type F-S-Like	Protein Coding
**LRPPRC**	Leucine Rich Pentatricopeptide Repeat Containing	Protein Coding
**LY96**	Lymphocyte Antigen 96	Protein Coding
**LYST**	Lysosomal Trafficking Regulator	Protein Coding
**MALAT1**	Metastasis Associated Lung Adenocarcinoma Transcript 1	RNA Gene
**MALT1**	MALT1 Paracaspase	Protein Coding
**MAPK14**	Mitogen-Activated Protein Kinase 14	Protein Coding
**MDM2**	MDM2 Proto-Oncogene	Protein Coding
**MEFV**	MEFV Innate Immuity Regulator, Pyrin	Protein Coding
**MEG3**	Maternally Expressed 3	RNA Gene
**MELK**	Maternal Embryonic Leucine Zipper Kinase	Protein Coding
**METTL14**	Methyltransferase 14, N6-Adenosine-Methyltransferase Subunit	Protein Coding
**METTL3**	Methyltransferase 3, N6-Adenosine-Methyltransferase Complex Catalytic Subunit	Protein Coding
**MIR103A1**	MicroRNA 103a-1	RNA Gene
**MIR103A2**	MicroRNA 103a-2	RNA Gene
**MIR107**	MicroRNA 107	RNA Gene
**MIR124-1**	MicroRNA 124-1	RNA Gene
**MIR125A**	MicroRNA 125a	RNA Gene
**MIR135B**	MicroRNA 135b	RNA Gene
**MIR139**	MicroRNA 139	RNA Gene
**MIR155**	MicroRNA 155	RNA Gene
**MIR15A**	MicroRNA 15a	RNA Gene
**MIR195**	MicroRNA 195	RNA Gene
**MIR204**	MicroRNA 204	RNA Gene
**MIR20B**	MicroRNA 20b	RNA Gene
**MIR21**	MicroRNA 21	RNA Gene
**MIR214**	MicroRNA 214	RNA Gene

**MIR22**	MicroRNA 22	RNA Gene
**MIR223**	MicroRNA 223	RNA Gene
**MIR23A**	MicroRNA 23a	RNA Gene
**MIR25**	MicroRNA 25	RNA Gene
**MIR30C1**	MicroRNA 30c-1	RNA Gene
**MIR4306**	MicroRNA 4306	RNA Gene
**MIR448**	MicroRNA 448	RNA Gene
**MIR455**	MicroRNA 455	RNA Gene
**MIR485**	MicroRNA 485	RNA Gene
**MIR497**	MicroRNA 497	RNA Gene
**MIR527**	MicroRNA 527	RNA Gene
**MIR556**	MicroRNA 556	RNA Gene
**MIR9-1**	MicroRNA 9-1	RNA Gene
**MIR9-2**	MicroRNA 9-2	RNA Gene
**MIR9-3**	MicroRNA 9-3	RNA Gene
**MKI67**	Marker Of Proliferation Ki-67	Protein Coding
**MLKL**	Mixed Lineage Kinase Domain Like Pseudokinase	Protein Coding
**MPEG1**	Macrophage Expressed 1	Protein Coding
**MRE11**	MRE11 Homolog, Double Strand Break Repair Nuclease	Protein Coding
**MST1**	Macrophage Stimulating 1	Protein Coding
**MYD88**	MYD88 Innate Immune Signal Transduction Adaptor	Protein Coding
**NAIP**	NLR Family Apoptosis Inhibitory Protein	Protein Coding
**NCR1**	Natural Cytotoxicity Triggering Receptor 1	Protein Coding
**NEAT1**	Nuclear Paraspeckle Assembly Transcript 1	RNA Gene
**NEDD4**	NEDD4 E3 Ubiquitin Protein Ligase	Protein Coding
**NEK7**	NIMA Related Kinase 7	Protein Coding
**NFE2L2**	NFE2 Like BZIP Transcription Factor 2	Protein Coding
**NFKB1**	Nuclear Factor Kappa B Subunit 1	Protein Coding
**NINJ1**	Ninjurin 1	Protein Coding
**NLRC4**	NLR Family CARD Domain Containing 4	Protein Coding
**NLRP1**	NLR Family Pyrin Domain Containing 1	Protein Coding
**NLRP13**	NLR Family Pyrin Domain Containing 13	Protein Coding
**NLRP3**	NLR Family Pyrin Domain Containing 3	Protein Coding
**NLRP6**	NLR Family Pyrin Domain Containing 6	Protein Coding
**NLRP7**	NLR Family Pyrin Domain Containing 7	Protein Coding
**NLRP9**	NLR Family Pyrin Domain Containing 9	Protein Coding
**NLRX1**	NLR Family Member X1	Protein Coding
**NOS1**	Nitric Oxide Synthase 1	Protein Coding
**NOS2**	Nitric Oxide Synthase 2	Protein Coding
**NR1H2**	Nuclear Receptor Subfamily 1 Group H Member 2	Protein Coding
**ORMDL3**	ORMDL Sphingolipid Biosynthesis Regulator 3	Protein Coding
**OSM**	Oncostatin M	Protein Coding
**P2RX7**	Purinergic Receptor P2X 7	Protein Coding
**PANX1**	Pannexin 1	Protein Coding
**PARP1**	Poly(ADP-Ribose) Polymerase 1	Protein Coding
**PCSK9**	Proprotein Convertase Subtilisin/Kexin Type 9	Protein Coding

**PDCD6IP**	Programmed Cell Death 6 Interacting Protein	Protein Coding
**PECAM1**	Platelet And Endothelial Cell Adhesion Molecule 1	Protein Coding
**PGF**	Placental Growth Factor	Protein Coding
**PKM**	Pyruvate Kinase M1/2	Protein Coding
**PKN2**	Protein Kinase N2	Protein Coding
**POLA1**	DNA Polymerase Alpha 1, Catalytic Subunit	Protein Coding
**POLA2**	DNA Polymerase Alpha 2, Accessory Subunit	Protein Coding
**POP1**	POP1 Homolog, Ribonuclease P/MRP Subunit	Protein Coding
**PPARG**	Peroxisome Proliferator Activated Receptor Gamma	Protein Coding
**PRDM1**	PR/SET Domain 1	Protein Coding
**PRF1**	Perforin 1	Protein Coding
**PRIM1**	DNA Primase Subunit 1	Protein Coding
**PRIM2**	DNA Primase Subunit 2	Protein Coding
**PRKN**	Parkin RBR E3 Ubiquitin Protein Ligase	Protein Coding
**PRMT5**	Protein Arginine Methyltransferase 5	Protein Coding
**PRTN3**	Proteinase 3	Protein Coding
**PTEN**	Phosphatase And Tensin Homolog	Protein Coding
**PTGS2**	Prostaglandin-Endoperoxide Synthase 2	Protein Coding
**PTPN11**	Protein Tyrosine Phosphatase Non-Receptor Type 11	Protein Coding
**PYCARD**	PYD And CARD Domain Containing	Protein Coding
**PYDC2**	Pyrin Domain Containing 2	Protein Coding
**RAB5A**	RAB5A, Member RAS Oncogene Family	Protein Coding
**RBBP4**	RB Binding Protein 4, Chromatin Remodeling Factor	Protein Coding
**RBBP7**	RB Binding Protein 7, Chromatin Remodeling Factor	Protein Coding
**RIPK1**	Receptor Interacting Serine/Threonine Kinase 1	Protein Coding
**RIPK3**	Receptor Interacting Serine/Threonine Kinase 3	Protein Coding
**RNF103-CHMP3**	RNF103-CHMP3 Readthrough	Protein Coding
**SCAF11**	SR-Related CTD Associated Factor 11	Protein Coding
**SDHB**	Succinate Dehydrogenase Complex Iron Sulfur Subunit B	Protein Coding
**SEC22B**	SEC22 Homolog B, Vesicle Trafficking Protein	Protein Coding
**SERPINB1**	Serpin Family B Member 1	Protein Coding
**SESN2**	Sestrin 2	Protein Coding
**SIGLEC14**	Sialic Acid Binding Ig Like Lectin 14	Protein Coding
**SIRT1**	Sirtuin 1	Protein Coding
**SLC16A4**	Solute Carrier Family 16 Member 4	Protein Coding
**SNIP1**	Smad Nuclear Interacting Protein 1	Protein Coding
**SQSTM1**	Sequestosome 1	Protein Coding
**STAT3**	Signal Transducer And Activator Of Transcription 3	Protein Coding
**STING1**	Stimulator Of Interferon Response CGAMP Interactor 1	Protein Coding
**STK4**	Serine/Threonine Kinase 4	Protein Coding
**STXBP2**	Syntaxin Binding Protein 2	Protein Coding
**STXBP3**	Syntaxin Binding Protein 3	Protein Coding
**SUZ12**	SUZ12 Polycomb Repressive Complex 2 Subunit	Protein Coding

**TCEA3**	Transcription Elongation Factor A3	Protein Coding
**TET2**	Tet Methylcytosine Dioxygenase 2	Protein Coding
**TFAM**	Transcription Factor A, Mitochondrial	Protein Coding
**TFAP2A**	Transcription Factor AP-2 Alpha	Protein Coding
**TLR2**	Toll Like Receptor 2	Protein Coding
**TLR3**	Toll Like Receptor 3	Protein Coding
**TLR8**	Toll Like Receptor 8	Protein Coding
**TLR9**	Toll Like Receptor 9	Protein Coding
**TNF**	Tumor Necrosis Factor	Protein Coding
**TNFSF13B**	TNF Superfamily Member 13b	Protein Coding
**TP53**	Tumor Protein P53	Protein Coding
**TP63**	Tumor Protein P63	Protein Coding
**TRAF6**	TNF Receptor Associated Factor 6	Protein Coding
**TREM1**	Triggering Receptor Expressed On Myeloid Cells 1	Protein Coding
**TREM2**	Triggering Receptor Expressed On Myeloid Cells 2	Protein Coding
**TRIM21**	Tripartite Motif Containing 21	Protein Coding
**TRIM24**	Tripartite Motif Containing 24	Protein Coding
**TRIM31**	Tripartite Motif Containing 31	Protein Coding
**TRPM2**	Transient Receptor Potential Cation Channel Subfamily M Member 2	Protein Coding
**TSLP**	Thymic Stromal Lymphopoietin	Protein Coding
**TUBB6**	Tubulin Beta 6 Class V	Protein Coding
**TXNIP**	Thioredoxin Interacting Protein	Protein Coding
**UBE2D2**	Ubiquitin Conjugating Enzyme E2 D2	Protein Coding
**UBE2D3**	Ubiquitin Conjugating Enzyme E2 D3	Protein Coding
**UBR2**	Ubiquitin Protein Ligase E3 Component N-Recognin 2	Protein Coding
**USF2**	Upstream Transcription Factor 2, C-Fos Interacting	Protein Coding
**USP24**	Ubiquitin Specific Peptidase 24	Protein Coding
**USP47**	Ubiquitin Specific Peptidase 47	Protein Coding
**UTS2**	Urotensin 2	Protein Coding
**VCAM1**	Vascular Cell Adhesion Molecule 1	Protein Coding
**VDR**	Vitamin D Receptor	Protein Coding
**VIM**	Vimentin	Protein Coding
**VPS28**	VPS28 Subunit Of ESCRT-I	Protein Coding
**VPS4B**	Vacuolar Protein Sorting 4 Homolog B	Protein Coding
**XIST**	X Inactive Specific Transcript	RNA Gene
**YWHAE**	Tyrosine 3-Monooxygenase/Tryptophan 5-Monooxygenase Activation Protein Epsilon	Protein Coding
**YWHAZ**	Tyrosine 3-Monooxygenase/Tryptophan 5-Monooxygenase Activation Protein Zeta	Protein Coding
**ZBP1**	Z-DNA Binding Protein 1	Protein Coding
**ZDHHC1**	Zinc Finger DHHC-Type Containing 1	Protein Coding
**ZFAS1**	ZNFX1 Antisense RNA 1	RNA Gene
